# Interacting Species Database (ISDB): comprehensive resource for interspecies interactions at the molecular level

**DOI:** 10.1093/bioinformatics/btag419

**Published:** 2026-06-25

**Authors:** Michael Mederer, Anupam Gautam, Oliver Kohlbacher, Andrei Lupas, Hadeer Elhabashy

**Affiliations:** Department for Internal Medicine I (Gastroenterology, Hepatology, Endocrinology, and Metabolism), Medical University of Innsbruck, Innsbruck A-6020, Austria; Institute for Bioinformatics and Medical Informatics, University of Tübingen, Maria-von-Linden-Str. 6, Tübingen 72076, Germany; Department of Microbiome Science, Max Planck Institute for Biology Tübingen, Max-Planck-Ring 5, Tübingen 72076, Germany; Institute for Bioinformatics and Medical Informatics, University of Tübingen, Maria-von-Linden-Str. 6, Tübingen 72076, Germany; Department of Computer Science, University of Tübingen, Maria-von-Linden-Str. 1, Tübingen 72076, Germany; Institute for Translational Bioinformatics, University Hospital Tübingen, Schaffhausenstraße 77, Tübingen 72072, Germany; Department of Protein Evolution, Max Planck Institute for Biology Tübingen, Max-Planck-Ring 5, Tübingen 72076, Germany; Department of Protein Evolution, Max Planck Institute for Biology Tübingen, Max-Planck-Ring 5, Tübingen 72076, Germany; Artificial Intelligence in Protein Science, University of Bayreuth, Universitätsstraße 30, Bayreuth 95447, Germany

## Abstract

**Motivation:**

Organisms within ecological systems often engage in molecular interactions that mediate key biological processes, such as protein–protein interactions involved in host–pathogen recognition and symbiosis. Characterization of these interactions at a molecular level is essential for understanding the mechanistic, evolutionary, and functional basis of interspecies interactions, as well as for informing potential therapeutic interventions. However, progress in this field is significantly impeded by the lack of a comprehensive database of interacting species at molecular resolution and the limited availability of experimental data.

**Results:**

We introduce the Interacting Species Database (ISDB), a comprehensive resource that catalogs interspecies interactions, annotated with NCBI taxonomic identifiers, interaction types and known molecular interactions. The ISDB encompasses 858 229 interacting species pairs and 171 713 interspecies protein-protein interactions within 261 287 organisms. ISDB is designed to support researchers in searching for, downloading, and depositing interspecies interaction data, which facilitates the study of ecological dynamics across diverse research domains.

**Availability and implementation:**

The ISDB is available via a web interface (https://www.elhabashylab.org/isdb), open-source code on GitHub (https://github.com/ElhabashyLab/ISDB) under the MIT license and is archived on Zenodo (Version v1.0.1, DOI: 10.5281/zenodo.20162385).

## 1 Introduction

Ecological interactions form the bedrock of biodiversity and ecosystem equilibrium, encompassing relationships such as predation, competition, mutualism, and parasitism (Brown *et al.* 2001, Wade 2007, Valiente-Banuet *et al.* 2015, Cable *et al.* 2017, Dixit 2024). Despite their importance, data on interactions between species are often fragmented and scattered across domain-specific databases. Many existing resources are limited by their focus on specific taxonomic groups or interaction types, reducing their utility for broader ecological analysis (Belbin *et al.* 2021, Del Ponte *et al.* 2021). In the absence of standardized formats for documenting ecological interactions, datasets often suffer from incomplete annotation or lack consistent nomenclature and/or unique identifiers, such as NCBI Taxonomic Identifiers (Taxon IDs), making it difficult to cross-reference data between sources (Fortuna *et al.* 2014, Poelen *et al.* 2014, Stephens *et al.* 2017). Additionally, data on interspecies interactions vary widely in resolution, with some resources providing only ecological relationships without molecular detail (Fortuna *et al.* 2014, Poelen *et al.* 2014), while others offer molecular insights but lack ecological context. Access to these datasets is further hindered by the absence of automated retrieval in some cases (Wardeh *et al.* 2015, Geiselman and Ember 2024), restrictions on researcher contributions (Guirimand *et al.* 2015, Lo Surdo *et al.* 2023), and the discontinuation of active maintenance (Guimaraes and Raimundo 2020). To date, no single resource integrates ecological and molecular interaction data across all taxonomic groups at a global scale, posing significant challenges for researchers seeking a holistic view of known ecological interactions.

To address these challenges and promote comprehensive research on interspecies interactions, we present the Interacting Species Database (ISDB)—a unified resource that integrates data from 21 distinct databases, with a particular focus on molecular-level interspecies interactions (Table 1). ISDB provides a comprehensive dataset of interspecies interactions, currently comprising 858 229 interacting species pairs between 261 287 organisms. Each interaction is annotated with key scientific details, including species scientific names, Taxon IDs, ontology identifiers, interaction types, and links to relevant publications and source databases. Moreover, ISDB provides molecular resolution for protein-protein interactions annotated with UniProt identifiers, whenever available, offering molecular insight into ecological interactions. ISDB supports comprehensive search functionality, data download, computational access, and data deposition, ensuring researchers can easily access and contribute to this resource.

**Table 1:** This table summarizes key characteristics of the datasets incorporated into ISDB, including the number of species, species pairs, and PPIs extracted from each source.^a^

Database	No. of species	No. of species pairs	No. of PPIs	No. of cross-referenced PPIs	Interaction types	Batch download	Database type	Citation
BioGRID	90	220	70 983	103 388	✓	✓	M	Stark *et al.* (2006)
IntAct	1260	1890	82 126	163 838	✓	✓	M	Del Toro *et al.* (2022)
MINT	482	658	17 624	54 976	✓	✓	M	Licata *et al.* (2012)
DIP	203	274	2064	84 434	✓	✓	M	Xenarios *et al.* (2000)
SIGNOR	8	7	81	69 438	✓	✓	M	Lo Surdo *et al.* (2023)
VirHostNet	293	347	36 225	75 488	✓	✓	HP/M	Guirimand *et al.* (2015)
PHISTO	588	587	46 115	65 523	✓	×	HP/M	Durmuş Tekir *et al.* (2013)
Interactomics	294	293	3976	84 670	✓	✓	HP/M	Teulière *et al.* (2023)
BV-BRC	140 365	152 996	0	9642	×	×	HP	Olson *et al.* (2022)
EID2	12 559	18 202	0	13 766	✓	✓	HP	Wardeh *et al.* (2015)
GMPD	1598	5683	0	1	×	×	HP	Stephens *et al.* (2017)
PHILM2web	422	1155	0	16 598	✓	×	HP	Le *et al.* (2022)
PHI-base	614	1179	0	4537	×	✓	HP	Urban *et al.* (2022)
HPIDB	734	967	0	53 134	✓	✓	HP	Ammari *et al.* (2016)
GloBI	124 879	684 288	0	333	✓	✓	E	Poelen *et al.* (2014)
Bat Eco-Interactions	3107	8099	0	0	✓	×	E	Geiselman and Ember (2024)
SIAD	3783	5104	0	2	✓	✓	E	Belbin *et al.* (2021)
IWDB	602	3533	0	0	×	✓	E	Guimaraes and Raimundo (2020)
Web of Life database	175	1096	0	0	×	✓	E	Fortuna *et al.* (2014); Ortega *et al.* (2022)
PIDA	619	804	0	0	×	✓	E	Bjorbækmo *et al.* (2020)
FGSCdb	19	15	0	0	✓	×	E	Del Ponte *et al.* (2021)

a The cross-referenced PPIs column indicates the number of curated PPIs available in ISDB for species pairs within each source database. The table also indicates whether each source database provides ontology interaction type classifications, reference citations, and batch download capabilities. The database type, indicating the primary focus of each resource, is categorized using the following identifiers: E (Ecological interactions), HP (Host–Pathogen interactions), and M (Molecular interactions).

## 2 Materials and methods

ISDB was developed using Python and Bash scripts and is available as open source code on GitHub (https://github.com/ElhabashyLab/ISDB) under MIT license. The GitHub repository also includes pre-built versions of the database in both CSV and TSV formats, released under the Creative Commons Attribution 4.0 International License, along with the source code for building and updating the database locally.

The ISDB web interface (https://www.elhabashylab.org/isdb) was developed using HTML, PHP and Bootstrap (version 5.3.3) and is hosted by the German Network for Bioinformatics Infrastructure (de.NBI). The underlying database is managed using MySQL (version 8.0.41-0ubuntu0.24.04.1) and PHP (version 8.3.6) to handle user queries.

### 2.1 Workflow

**Figure 1:**
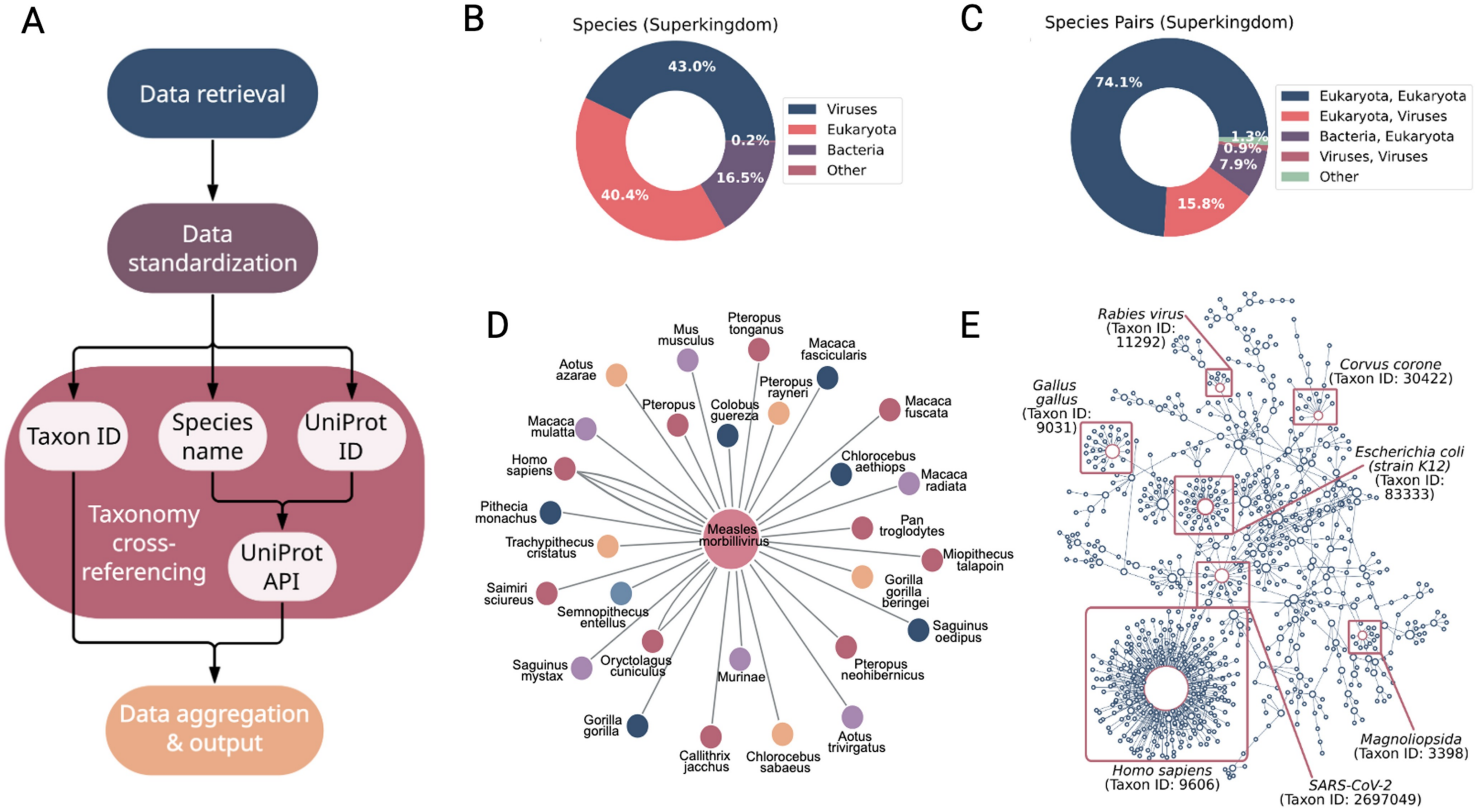
(A) Flowchart outlining the key steps in ISDB creation, including data retrieval, standardization, taxonomy annotation, and aggregation. (B) Ring diagram illustrating the proportional distribution of species by superkingdom, (C) Ring diagram illustrating the classification of species interactions according to superkingdom, and (D) Interspecies interaction network of Measles morbillivirus (Taxon ID: 11 234) as retrieved from the ISDB web interface, showing 28 documented host organisms. Multiple edges between two nodes reflect the presence of protein–protein interaction (PPI) entries describing their interaction. (E) An example interspecies interaction network retrieved from ISDB, where each node represents a species (labeled with its scientific name and NCBI Taxon ID) and each edge represents a documented interaction between two species.

The ISDB build process consists of four steps (Fig. 1A): data retrieval, data standardization, taxonomy cross-referencing, and data aggregation and output.

**Data retrieval.** ISDB consolidates species interaction data, including protein-protein interactions, from a variety of freely accessible resources, which can be categorized into three principal groups: ecological databases, host-pathogen databases, and molecular interaction databases. Ecological databases [e.g. Web of Life (Fortuna *et al.* 2014) and the Bat Eco-Interactions database (Geiselman and Ember 2024)] capture interactions between species within natural ecosystems, covering a wide range of biological relationships such as predation and co-habitation. Host-pathogen databases [e.g. VirHostNet (Guirimand *et al.* 2015) and PHILM2web (Le *et al.* 2022)], on the other hand, specialize in disease-causing species interactions covering a diverse set of virus-host, bacteria-host, and parasite-host dynamics. Molecular databases [e.g. IntAct (Del Toro *et al.* 2022) and DIP (Xenarios *et al.* 2000)] offer a more granular view, providing data on molecular interactions among proteins. Table 1 presents an overview of the sources integrated into ISDB, highlighting their specific contributions in terms of interspecies interactions and molecular-level data, along with relevant citations and key attributes.

**Data standardization.** For each source, data is standardized into a generic column-based format, and available interaction annotations are extracted. This includes interaction types, ontology identifiers, and references, ensuring consistent data representation across diverse sources.

**Taxonomy cross-referencing.** ISDB ensures consistency in cross-referencing interactions from diverse sources by using NCBI Taxon IDs as standardized identifiers. When a Taxon ID is unavailable, species names are queried through the UniProt Taxonomy service, using either the species *scientific name* or *common name* fields (UniProt Consortium 2023). Alternatively, if the UniProt accession number of a protein is available, the UniProt API service is used to retrieve the corresponding Taxon ID (Nightingale *et al.* 2017).

**Data aggregation and output.** The final stage of data processing involves refining the aggregated data by removing redundancies in Taxon IDs and UniProt IDs. Redundant records are consolidated into single entries while preserving the union of interaction type annotations, ontology identifiers, literature references, and source database information. The curated results are then exported in both CSV and TSV formats. The processed database provides a comprehensive set of information, including Taxon IDs, species scientific names, UniProt accession, ontology identifiers, interaction types, relevant publications, and the source databases.

### 2.2 Execution and dependencies

ISDB requires Python version 3.11.7 and several Python libraries, including Pandas 2.0.3, NumPy 1.24.3, and Requests 2.31.0. To compile the database locally, users can execute the main build script:

bash buildDB.sh

The script automatically downloads and parses most data sources; however, several datasets must be downloaded manually from their respective websites prior to execution. Users can configure multiple build parameters, including options to overwrite existing files, retain or remove intermediate data, and integrate user-contributed datasets. Detailed installation and maintenance instructions are provided in the GitHub documentation to support local deployment and customization of ISDB.

## 3 Results

We developed ISDB as a centralized platform for ecological interaction data enriched with molecular-level protein–protein interaction (PPI) information. ISDB currently contains 858 229 unique pairwise species interactions involving 261 287 distinct organisms, with a total size of 23 MB (compressed TSV/CSV). These species span multiple superkingdoms (domains), including eukaryotes (40.4%), viruses (42.9%), bacteria (16.5%), and others (0.2%) (Fig. 1B). The most represented species include *Homo sapiens* (15.58%), *Mus musculus* (2.85%), and *Severe acute respiratory syndrome coronavirus* (1.44%). Interspecies interactions are categorized by superkingdom associations and are dominated by eukaryote–eukaryote interactions (74.1%), followed by virus–eukaryote (15.8%) and bacteria–eukaryote (7.9%) interactions (Fig. 1C), enabling comparative analyses across diverse ecological and host–pathogen systems. Figure 1E illustrates a representative ecological interaction network within ISDB, where nodes correspond to species and edges denote documented interspecies associations.

ISDB comprises 171 713 curated interspecies PPIs across 1982 species pairs (0.23% of all species pairs) and 1362 species (0.52% of all species). The high density of PPIs among a limited subset of species pairs reflects a bias toward extensively studied host–pathogen systems, particularly human-associated viruses, and highlights the comparatively limited molecular characterization of the majority of ecological interactions. We cross-referenced all interspecies interaction records with these PPIs to quantify the extent of molecular annotation within the database (Table 1). Molecular annotations were substantially more prevalent in host–pathogen interaction resources than in broader ecological interaction resources. Interaction types in ISDB are represented by 430 curated keywords and ontology identifiers inherited from the constituent resources, providing broad coverage of heterogeneous interaction categories. Future releases of ISDB will prioritize a standardized interaction type schema to improve consistency, interoperability, and downstream computational usability.

ISDB supports bulk download through the GitHub repository and web server, and the current release is also provided as Supplementary Material with this work. All ISDB build scripts are openly available on GitHub under the MIT license, enabling users to independently rebuild and deploy local ISDB instances. The ISDB web interface supports queries using species names, NCBI Taxon identifiers, or UniProt accessions, and search results can be downloaded in CSV or TSV formats for downstream analysis. Users may also contribute data by submitting interacting species or protein pairs with supporting literature references. Submitted entries are validated through manual curation prior to incorporation into the database.

To demonstrate the practical utility of ISDB, we queried *Measles morbillivirus* (Taxon ID: 11 234). ISDB returned an interspecies interaction network spanning 28 host species, including *Homo sapiens* (human; Taxon ID: 9606), *Pan troglodytes* (chimpanzee; Taxon ID: 9598), and *Pteropus tonganus* (Pacific flying fox; Taxon ID: 77 217), among others (Fig. 1D). In addition, ISDB returned 13 curated PPIs between *Measles morbillivirus* proteins and host proteins. These included 10 interactions with *Homo sapiens*, 2 with *Chlorocebus aethiops* (green monkey; Taxon ID: 9534), and 1 with *Saguinus oedipus* (cotton-top tamarin; Taxon ID: 9490). These interactions were integrated from IntAct, DIP, PHISTO, and VirHostNet, along with supporting literature indexed in PubMed. This unified framework streamlines the exploration of molecular interactions across species and enables hypothesis-driven identification and testing of homologous interacting proteins across phylogenetically diverse species.

## 4 Discussion

Comprehensive species interaction data are fundamental for advancing ecological and evolutionary research, as well as for understanding complex ecosystems and biodiversity (Lawrence *et al.* 2012, Hardisty *et al.* 2013, Toju *et al.* 2017). However, despite significant efforts to compile such data, existing records remain fragmented across literature and specialized databases, hindering cross-domain ecological analyses. ISDB addresses this challenge by integrating data from 21 sources, creating a unified platform that enhances accessibility and facilitates broader ecological research.

A critical aspect of effective data integration is standardization. Inconsistencies in data formats, species nomenclature, and the lack of unified taxonomic identifiers have historically posed significant obstacles to knowledge accumulation and interoperability (Helmy *et al.* 2016). ISDB mitigates these issues by incorporating NCBI taxonomic identifiers and standardized scientific names, thereby ensuring precise species identification and enabling seamless connections to genomic and proteomic data (Sayers *et al.* 2022, UniProt Consortium 2023).

Despite these standardization efforts, challenges remain in classifying interaction types. ISDB currently uses a diverse set of 430 terms derived from both keyword-based and ontology-driven classifications. However, variations in terminology introduce redundancy and ambiguity, as similar or overlapping interaction types may be categorized under different terms. For instance, the broad term “association” encompasses multiple interaction types, reducing interpretability. Additionally, the directionality of interactions remains undefined in a systematic manner, necessitating further refinement and manual curation. Establishing a unified global nomenclature for interaction types is essential to improve data integration, consistency, and usability (Hardisty *et al.* 2013, Wood *et al.* 2022).

Beyond ecological classifications, many species interactions—such as pathogen–host relationships—are driven by macromolecular contacts, primarily involving proteins (Farnsworth *et al.* 2017, Defranoux and Mollo 2020). However, fewer than 0.3% of the species interactions recorded in ISDB currently include molecular-resolution data. Future development of ISDB should prioritize the integration of molecular-level descriptions. This includes not only interactions between proteins, but also with other biomolecules (e.g. RNA, metabolites, or small molecules), alongside richer metadata to boost the database’s utility across multiple research domains.

Another limitation is the under-representation of specific ecological interactions. For instance, interactions involving humans are by far the most represented in the ISDB due to the emphasis of biomedical research on human health. Conversely, bacterial-bacterial and bacterial-viral interactions are vastly underrepresented, likely due to biases in data collection and inherent difficulties in systematically detecting and documenting microbial interactions. Addressing these gaps will require targeted data acquisition efforts and the integration of high-throughput experimental datasets.

Finally, the long-term sustainability and accessibility of ecological databases remain important challenges, often limited by inconsistent maintenance and restricted reproducibility. ISDB addresses these challenges through biannual releases, openly available build and analysis scripts under the MIT license, and distribution of prebuilt database versions through the GitHub repository, web server, and Supplementary Material. The codebase is also permanently archived through Zenodo (DOI: 10.5281/zenodo.2,01,62,385). Long-term maintenance and curation of ISDB are supported by the authors and members of the Elhabashy Lab, ensuring continued development and accessibility of the resource. In summary, ISDB represents a significant step forward in unifying interspecies interaction data across diverse sources, enhancing accessibility and interoperability. While challenges in terminology, molecular resolution, and microbial interaction coverage remain, continued refinement and expansion will further strengthen ISDB’s role as a critical resource for ecological and molecular research.

## 5 Conclusion

ISDB provides a comprehensive platform for studying interspecies interactions by integrating data from 21 distinct sources and bridging ecological and molecular perspectives. By standardizing species names, taxonomic identifiers, and molecular interaction data—particularly protein-protein interactions—ISDB mitigates challenges associated with data fragmentation and inconsistency across specialized databases. Through its intuitive interface, open-source framework, and computational accessibility, ISDB holds significant potential to expand into a dynamic community-driven resource and support diverse applications in biodiversity conservation, disease management, and evolutionary biology.

## Supplementary Material

btag419_Supplementary_Data

## Data Availability

The Interacting Species Database (ISDB) is freely available in CSV and TSV formats under the Creative Commons Attribution 4.0 International License. It can be accessed via the web interface (https://www.elhabashylab.org/isdb) and the GitHub repository (https://github.com/ElhabashyLab/ISDB). The version released at the time of publication is also provided as Supplementary Material to this article. All build scripts are available as open-source code on GitHub (https://github.com/ElhabashyLab/ISDB) under the MIT License and are permanently archived on Zenodo (Version v1.0.1, DOI: 10.5281/zenodo.20162385).
